# Rapid Detection of *Bacillus subtilis* via RPA Combined with CRISPR/Cas12a

**DOI:** 10.3390/foods15081419

**Published:** 2026-04-18

**Authors:** Qingchao Xie, Wei Wu, Pengju Zhao, Yang Yuan, Hongmin Zhang, Yong Zhao

**Affiliations:** 1 College of Food Science and Technology, Shanghai Ocean University, Shanghai 201306, China; qcxie@shou.edu.cn (Q.X.); 17639388182@163.com (W.W.); zpjgosick@163.com (P.Z.); 17784950862@163.com (Y.Y.); 2 Laboratory of Quality & Safety Risk Assessment for Aquatic Product on Storage and Preservation (Shanghai), Ministry of Agriculture and Rural Affairs, Shanghai 201306, China

**Keywords:** *Bacillus subtilis*, rapid detection, CRISPR/Cas12a, recombinase polymerase amplification

## Abstract

*Bacillus* and *Paenibacillus* species are common and widely distributed microorganisms in food systems, often implicated in food spoilage and quality issues. *Bacillus subtilis*, in particular, has been associated with gas production and package bulging in seasoned foods. In this study, we developed a rapid and visual detection method for *Bacillus subtilis* by integrating (Recombinase Polymerase Amplification) RPA with (Clustered Regularly Interspaced Short Palindromic Repeats) CRISPR/Cas12a technology (designated as RPA-CRISPR/Cas12a). Specific RPA primers and probes were designed based on the conserved *gyrB* gene of *Bacillus subtilis*. Two sets of crRNA were designed according to the number of T-rich PAM sites on the RPA-amplified target sequence, and the reaction conditions were optimized in combination with the CRISPR/Cas12a trans-cleavage detection technology. Under optimized conditions, the crRNA3 guide (with a TT-rich PAM site) demonstrated superior cleavage efficiency compared to crRNA2 (TTT-rich PAM), while crRNA1 (TTTT-rich PAM) showed no activity. The assay achieved a detection limit of 150 pg/μL for genomic DNA and 5.5 CFU/mL for bacterial suspensions within 10 min at 37 °C. The method exhibited high specificity and sensitivity, providing a robust tool for early and on-site detection of *Bacillus subtilis* in food products.

## 1. Introduction

*Bacillus subtilis* (BS), a ubiquitous Gram-positive bacterium, is valued in the food industry for its probiotic properties, enzyme production, and role in fermentation processes such as lactic acid beverage production and the utilization of brewer’s spent grain [1,2,3]. While generally regarded as safe, its anaerobic metabolism can lead to gas production and spoilage in products like seasoned foods and stinky tofu, resulting in significant economic losses [4]. This dual nature underscores the need for rapid and accurate detection methods to ensure product quality and safety. Conventional techniques, including culture-based methods, immunoassays, and PCR, are often time-consuming, labor-intensive, or prone to false positives/negatives [5]. Although emerging approaches like Raman spectroscopy have shown promise in detecting bacterial spores, there remains a need for simpler, faster, and more reliable detection platforms that are suitable for on-site application without compromising accuracy.

Molecular biological methods that directly target microbial nucleic acids are among the most sensitive and rapidly advancing detection approaches. In particular, isothermal amplification techniques offer high sensitivity, simpler nucleic acid preparation compared with real-time quantitative PCR, and allow rapid result acquisition within a short time. For the accurate identification of bacterial species with highly conserved homologous sequences, the construction of phylogenetic trees based on a combination of 16SrRNA and *gyrB* gene sequences has been proven effective for distinguishing *Bacillus amyloliquefaciens* and related species [6]. Multiple genes, including 16SrRNA, *rpoB*, *gyrA*, and *gyrB*, have been widely used for the detection and identification of *Bacillus* species [7,8]. However, the 16SrRNA gene exhibits high sequence similarity within the genus *Bacillus*, making it insufficient to distinguish closely related species. In contrast, the *gyrB* gene shows greater interspecies divergence and has been validated as a reliable phylogenetic marker for differentiating BS from its closely related taxa [9]. Accordingly, the *gyrB* gene, which possesses conserved regions across genera but sufficient variability between species, was selected as the target gene in this study. Using this target, the developed method could specifically identify BS without cross-reactivity with other tested pathogenic bacteria.

CRISPR is an adaptive immune system in bacteria and archaea that defends against viral invasions. It has now evolved into a powerful gene-editing tool in genetic engineering, capable of rapidly and accurately identifying and cleaving specific nucleic acid sequences to defend against mobile genetic elements [10,11,12]. The discovery of Cas12a has facilitated the development of DNA detection methods such as DETECTR [13] and HOLMES [10]. The Cas12a family comprises a class of nucleases regulated by a single crRNA capable of specifically recognizing and cleaving dsDNA containing Protospacer Adjacent Motif (PAM) auxiliary sequences (5′-TTTN-3′ or 5′-TTN-3′). When CRISPR/Cas12a is guided by crRNA to target dsDNA, it can cleave ssDNA, making it a specific tool for dsDNA detection [14,15]. PAM recognition is a critical step in identifying and degrading DNA molecules, as it allows the CRISPR/Cas system to distinguish between self-genomic DNA and foreign nucleic acids [16,17]. In summary, CRISPR detection involves the activation of the trans-cleavage activity of Cas proteins by target nucleic acids, which cleave fluorescently labeled probes in the system, generating a fluorescent signal and thereby completing the detection of target nucleic acids [18]. Integrating CRISPR/Cas12a with RPA mitigates these issues by introducing an additional layer of sequence-specific verification, thereby enhancing detection specificity and reducing false positives.

In this study, we developed a two-step RPA-CRISPR/Cas12a assay targeting the conserved *gyrB* gene of BS. This method separates amplification from detection to minimize false positives and provides a visual readout, offering a rapid, accurate, and equipment-minimized platform for on-site detection of BS in food products.

## 2. Materials and Methods

### 2.1. Reagents and Instruments

BS ATCC6633, *Bacillus cereus* LA10, and common pathogenic strains including *Escherichia coli* EC-1, *Salmonella* S-31, *Vibrio parahaemolyticus* VP28, *Listeria monocytogenes* LM16, and *Staphylococcus aureus* ATCC25922 were all preserved in our laboratory.

TIANamp Bacteria DNA Kit (DP302) (Tiangen Biochemical Technology, Beijing, China); RPA Basic Isothermal Amplification Kit (Weifang Amplfuture Biotechnology, Weifang, Shandong, China); Nuclease-free water; Phenol-Chloroform-Isoamyl Alcohol (25:24:1) DNA extraction solution (Sangon Biotech, Shanghai, China); DNA-Grade CTAB Solution; 50× TAE buffer (Sangon Biotech, Shanghai, China); Agarose; Nucleic acid gel stain; 6× DNA Loading Buffer; Absolute ethanol; Isopropanol; TranscriptMax T7 High Yield RNA Synthesis Kit (ToloBio Biotechnology, Shanghai, China); LbCas12a (Cpf1) Nuclease (ToloBio Biotechnology, Shanghai, China); HOLMES ssDNA Reporter (FAM) (ToloBio Biotechnology, Shanghai, China). When the HOLMES ssDNA reporter probe is intact, the fluorescence signal emitted by the reporter group is quenched. When the probe is cleaved, the FAM reporter group at the 5′ end separates from the quencher, resulting in a detectable fluorescence signal.

Constant temperature shaker (Guansen Biotechnology, Shanghai, China); Refrigerated centrifuge (Eppendorf, Hamburg, Germany); Gel imaging system; Constant temperature metal bath (Bioer Technology, Hangzhou, China); Real-Time PCR System; DYY-6D electrophoresis apparatus (Beijing Liuyi Instrument Factory, Beijing, China); T6 New Century UV-Vis spectrophotometer (Beijing, China); NanoDrop One ultra-micro spectrophotometer (Thermo Fisher Scientific, Waltham, MA, USA); Fluorescence reader.

### 2.2. Strain Activation and Bacterial Culture Preparation

Brain Heart Infusion (BHI) medium (200 mL) was prepared and sterilized in an autoclave at 121 °C for 20 min. For primary activation, 200 μL of BS strain preserved at −80 °C was inoculated into 5 mL of medium and incubated in a shaker at 180 rpm and 37 °C for 6 h. For secondary activation, 200 μL of the primary culture was transferred into 5 mL of fresh medium and incubated overnight (12 h) at 180 rpm and 37 °C. Then, 1 mL of the bacterial suspension was centrifuged at 13,000× *g* for 3 min to collect the bacterial cells. Genomic DNA was extracted using a DNA extraction kit (spin column type) according to the manufacturer’s instructions. The concentration of the extracted DNA was measured using a NanoDrop One ultra-micro spectrophotometer (A260/A280 ratio between 1.8 and 2.2). The DNA was labeled and stored at −20 °C for future use.

The concentration of the BS bacterial suspension was determined to be 5.5 × 10^9^ CFU/mL using plate colony counting. The BS suspension was serially diluted to the required concentrations (ranging from 5.5 × 10^6^ CFU/mL to 5.5 CFU/mL). DNA was extracted using the boiling method for detecting bacterial suspension sensitivity: 1 mL of each diluted bacterial suspension was centrifuged at 13,000× *g* for 1 min, the supernatant (medium) was discarded, and 100 μL of sterile ultrapure water was added to thoroughly resuspend the bacterial pellet. The mixture was heated at 95 °C for 10 min, immediately centrifuged at ≥13,000× *g* for 10–15 min, and the supernatant was carefully transferred to a new sterile 1.5 mL centrifuge tube using a pipette. The extracted DNA was stored at −20 °C.

### 2.3. RPA and crRNA Primer Design

The target gene sequences of BS were retrieved and aligned for conserved region analysis using the National Center for Biotechnology Information (NCBI) database (https://www.ncbi.nlm.nih.gov/). Based on the identified highly conserved sequences, specific RPA primers were designed accordingly. The length of the designed RPA primers was set at 30–35 bp, and the size of the amplified target fragment was controlled within 150–500 bp to ensure efficient and specific isothermal amplification. During primer design, we used consecutive repetitive bases and secondary structures. All primers were designed with the assistance of Primer Premier 5.0 software (Premier Biosoft, San Francisco, CA, USA). The optimal primer pair for RPA amplification was determined by agarose gel electrophoresis, and the specific primer combination used in this study is clearly listed in Table S1.

**Figure 1:**
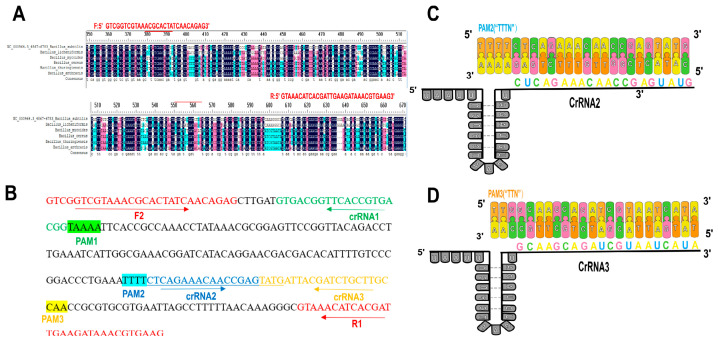
(**A**) Screening of the optimal amplification primer pair using RPA technology. Sequence comparison across different Bacillus species was performed to ensure specificity. (**B**) Design locations of the RPA primers and crRNAs. (**C**) Schematic diagram of dsDNA target detection by Cas12a/crRNA2. (**D**) Schematic diagram of dsDNA target detection by Cas12a/crRNA3.

Based on the characteristics of the guide crRNA target sequence bound by Cas12a, the PAM sites recognized by Cas12a were identified. crRNAs were designed targeting the RPA-amplified products of the *gyrB* gene. Since Cas12a prefers editing regions enriched with TTTN (N represents any nucleotide) or TTTV (V = A/C/G) in the genome, three crRNAs were designed according to the thymine-rich regions to determine the most efficient cleavage crRNA. The design of primers and crRNAs considered two main aspects: First, the crRNA sequence must target the conserved region amplified by RPA, and the target sequence length should not be too long—generally 200–300 bp is sufficient. Second, the crRNA sequence must not overlap with the RPA primers. The final crRNA was selected based on fluorescence curve analysis. All primers were synthesized by Sangon Biotech (Shanghai) Co., Ltd. After synthesis, the transcription primers were used to transcribe DNA into crRNA following the instructions of the TranscriptMax T7 High Yield crRNA Synthesis and Purification Kit. The concentration of the synthesized crRNA was measured using a NanoDrop One ultra-micro spectrophotometer (A260/A280 ratio between 1.8 and 2.2). The crRNA was synthesized and diluted to a working concentration of 1 μM. Three crRNAs targeting BS were designed. All crRNAs contained a conserved 20-nucleotide (nt) repeat sequence (UAAUUUCUACUAAGUGUAGAU) required for LbCas12a recognition, followed by a target-specific spacer sequence complementary to the genomic DNA of BS Table 1. Figure 1 visually illustrates the design locations of the RPA primers and crRNA.

### 2.4. RPA-CRISPR/Cas12a Detection Method

The RPA amplification reaction system (50 μL) was prepared as follows: 29.4 μL of A buffer, 2 μL of forward primer (10 μmol/L), 2 μL of reverse primer (10 μmol/L), 2 μL of BS genomic DNA, and 12.1 μL of ddH_2_O were successively added to a lyophilized powder tube. Then, 2.5 μL of B buffer was added to the reaction mixture, thoroughly mixed, and centrifuged. The mixture was immediately incubated in a constant temperature metal bath at 38–40 °C for 15–30 min. After amplification, an equal volume of Phenol-Chloroform-Isoamyl Alcohol (25:24:1) was added to purify the RPA products by centrifugation at 12,000 rpm for 3 min. The supernatant obtained after centrifugation contained the purified RPA products, which were subsequently analyzed using 2% agarose gel electrophoresis (AGE).

The CRISPR/Cas12a protein-mediated cleavage reaction system (20 μL) consisted of: 2 μL of 10× Cas12a reaction buffer, 2 μL of crRNA, 1 μL of Cas12a nuclease (1 μM), 1 μL of reporter ssDNA (1 μM), 1 μL of RPA amplification product, and 13 μL of nuclease-free water. The entire system was prepared on ice. For real-time fluorescence quantitative detection via RPA-CRISPR/Cas12a, the reaction mixture was thoroughly mixed, briefly centrifuged, and then placed in a qPCR instrument. The reaction was carried out at 37 °C for 40 cycles, with signals collected every 30 s.

### 2.5. Optimization of Reaction Conditions for RPA-CRISPR/Cas12a Assay

**Figure 2:**
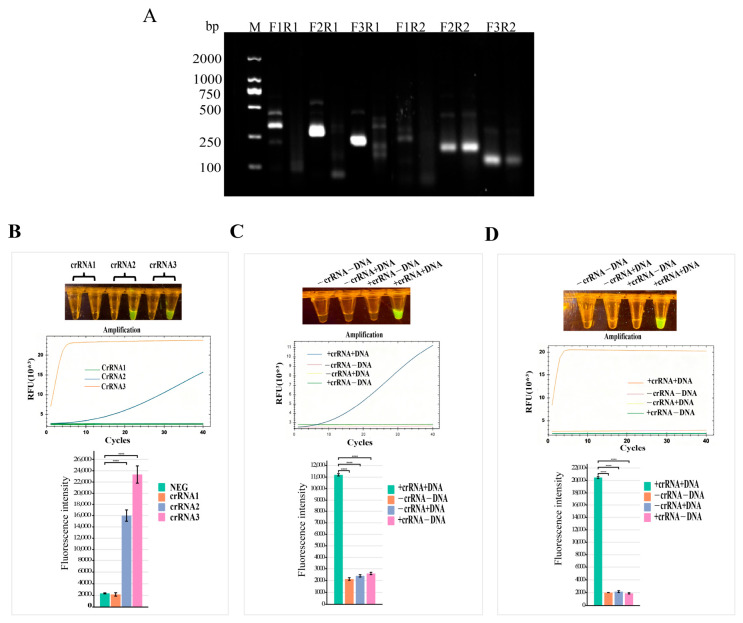
Optimization of the RPA-CRISPR/Cas12a assay for BS detection. (**A**) Gel electrophoresis of RPA primer screening. (**B**) crRNA screening via fluorescence visualization, real-time amplification, and end-point fluorescence. (**C**) Optimization of primer-crRNA combinations. (**D**) Validation of the final optimized system. Data are mean ± SD (n = 3). **** indicates a statistically significant difference with *p* < 0.0001.

The feasibility of the detection system was verified using transcribed crRNA. A 1 μL aliquot of the RPA amplification product was added to the CRISPR/Cas12a detection system. Preliminary experiments confirmed that this method could detect BS (Figure 2B) and identified effective crRNAs. Based on these results, the amounts of crRNA and Cas12a nuclease in the detection system were optimized.

- crRNA (1 μM) dosage screening: Different volumes of crRNA were tested: 0 μL, 1 μL, 2 μL, and 4 μL. The optimal crRNA dosage was determined based on fluorescence curves and intensity.
- Cas12a (1 μM) protein dosage screening: Different volumes of Cas12a protein were tested: 0 μL, 1 μL, 2 μL, and 4 μL. The optimal Cas12a dosage was determined based on fluorescence curves and intensity.

After preparation, the reaction mixture was mixed, briefly centrifuged, and immediately transferred to a real-time fluorescence PCR instrument. The reaction was performed at 37 °C for 40 cycles, with signals collected every 30 s. The optimal crRNA and Cas12a protein dosages were selected based on the fluorescence values obtained from the qPCR fluorescence curves.

- Reaction temperature: Based on the optimal RPA reaction conditions and system, five reaction temperatures were tested for CRISPR/Cas12a detection: 5 °C, 25 °C, 30 °C, 37 °C, and 45 °C. The CRISPR reaction time was set to 10 min, with nuclease-free water used as a blank control.
- Reaction time: Based on the optimal RPA reaction conditions and system, six reaction times were tested for CRISPR/Cas12a detection: 5, 10, 15, 20, 30, and 40 min. The CRISPR reaction temperature was set to 37 °C, with nuclease-free water used as a blank control.

### 2.6. Specificity Evaluation of the RPA-CRISPR/Cas12a Detection System

Based on the optimized reaction temperature and time for the RPA-CRISPR/Cas12a detection system, the specificity of the crRNA was evaluated using visual fluorescence detection. Genomic DNA from seven representative strains was used, including BS, the closely related species *Bacillus cereus* within the Bacillus genus, and several common foodborne pathogens, including *Escherichia coli*, *Salmonella*, *Vibrio parahaemolyticus*, *Staphylococcus aureus*, and *Listeria monocytogenes*.

Among these, Bacillus cereus was selected as a phylogenetically close relative within the Bacillus genus, which is frequently encountered in food matrices and shares high genomic similarity with BS, making it a representative and critical control for verifying species specificity. The other strains included common foodborne pathogens from different genera to exclude cross-reactivity with unrelated microorganisms. Together, this panel allows comprehensive evaluation of the target-specific recognition capability of the established assay for BS.

### 2.7. Sensitivity Analysis of the RPA-CRISPR/Cas12a Detection System

The sensitivity for genomic DNA detection was evaluated. The purified genomic DNA of BS was serially diluted 10-fold to obtain concentrations of 150 ng/μL, 15 ng/μL, 1.5 ng/μL, 150 pg/μL, 15 pg/μL, 1.5 pg/μL, and 150 fg/μL. These diluted amplification products were used as templates.

For the detection sensitivity of pure BS suspension, logarithmic-phase cultures were serially 10-fold diluted. DNA was extracted by the boiling method and used as the template for RPA-CRISPR/Cas12a amplification. The detection limit and performance of the established method were compared with the specifications outlined in GB/T 26428-2010 [19] Method for determination of *Bacillus subtilis* in feeds and the general microbial counting requirements specified in GB 4789.2-2022 [20] National food safety standard Food microbiological examination: Aerobic plate count.

#### Definition of Limit of Detection (LOD)

LOD was defined as the lowest concentration of BS that generated a fluorescence signal significantly higher than the background noise. It was calculated based on the standard deviation (SD) of the negative control (NEG) signals using the 3-sigma rule (3σ):

LOD = Mean_NEG_ + 3 × SD_NEG_ (1)

Mean_NEG_ is the average fluorescence value of the negative control group and SD_NEG_ is the corresponding standard deviation. SD was determined as 10 RFU. Additionally, linear regression analysis was performed between the log-transformed CFU/mL values and the cycle threshold (Ct) values to establish the standard curve, and the LOD was verified based on the intersection of the regression line with the LOD threshold.

### 2.8. Quantitative Analysis of Gel Electrophoresis and Fluorescence Images

The amplification efficiency of RPA products was quantitatively evaluated by measuring the grayscale intensity of target DNA bands on agarose gel electrophoresis images using ImageJ 1.53k softwaresoftware (National Institutes of Health, Bethesda, MD, USA). For fluorescence-based detection, fluorescence signals were collected using a fluorescence quantitative PCR system and analyzed analogously with ImageJ. For each sample, the average grayscale intensity was calculated after background subtraction. Relative amplification efficiency was expressed as the normalized grayscale intensity or normalized fluorescence intensity. The quantified data were plotted as bar graphs using GraphPad Prism 9.0 software (GraphPad Software, San Diego, CA, USA). All experiments were performed in triplicate, and data are presented as mean ± SD. Statistical comparisons were performed using one-way ANOVA followed by Tukey’s multiple comparison test, with **** indicating *p* < 0.0001.

### 2.9. Statistical Analysis

All experiments were performed in triplicate (n = 3) with independent biological replicates. Data are presented as mean ± SD. Statistical analyses were performed using GraphPad Prism 9.0 software. For comparisons of fluorescence intensity between different groups (e.g., different crRNAs, primer combinations), one-way analysis of variance ANOVA followed by Tukey’s multiple comparison test was used to determine significant differences. A *p*-value of < 0.05 was considered statistically significant, with **** indicating *p* < 0.0001, *** indicating *p* < 0.001, ** indicating *p* < 0.01, and * indicating *p* < 0.05.

## 3. Results

### 3.1. Design and Screening of RPA and crRNA Primers

This study selected the *gyrB* gene as the detection target, which exhibits high conservation within the genus *Bacillus* but sufficient interspecies divergence to distinguish closely related species. The established assay specifically identified BS while showing no cross-reactivity with other tested bacterial strains, including common foodborne pathogens and the closely related species *Bacillus cereus*.

To establish a highly sensitive and specific RPA-CRISPR/Cas12a assay for BS detection, we first optimized the RPA primer pairs. As shown in Figure 2A, six primer combinations (F1R1, F2R1, F3R1, F1R2, F2R2, F3R2) were initially screened via agarose gel electrophoresis. Among them, F1R1, F2R1, F3R1, and F2R1 produced clear target bands, while F2R2 and F3R2 showed non-specific amplification or weak signals. Based on band brightness and specificity, F2R1 was selected as the optimal RPA primer pair for subsequent experiments.

Using RPA products obtained under the optimal reaction time of 15 min as templates, crRNAs for the CRISPR/Cas12a detection system were screened. crRNA1, crRNA2, and crRNA3 were introduced into the reaction system separately. No fluorescence signal was observed with crRNA1, while the fluorescence intensity of the crRNA3 group was higher than that of the crRNA2 group, as shown in Figure 2B. Feasibility tests were further conducted for crRNA2 and crRNA3. Figure 2C,D demonstrate that fluorescence was only activated when both crRNA and DNA were present in the system. Both crRNA2 and crRNA3 were capable of detecting BS.

### 3.2. Optimization of RPA Reaction Conditions

**Figure 3:**
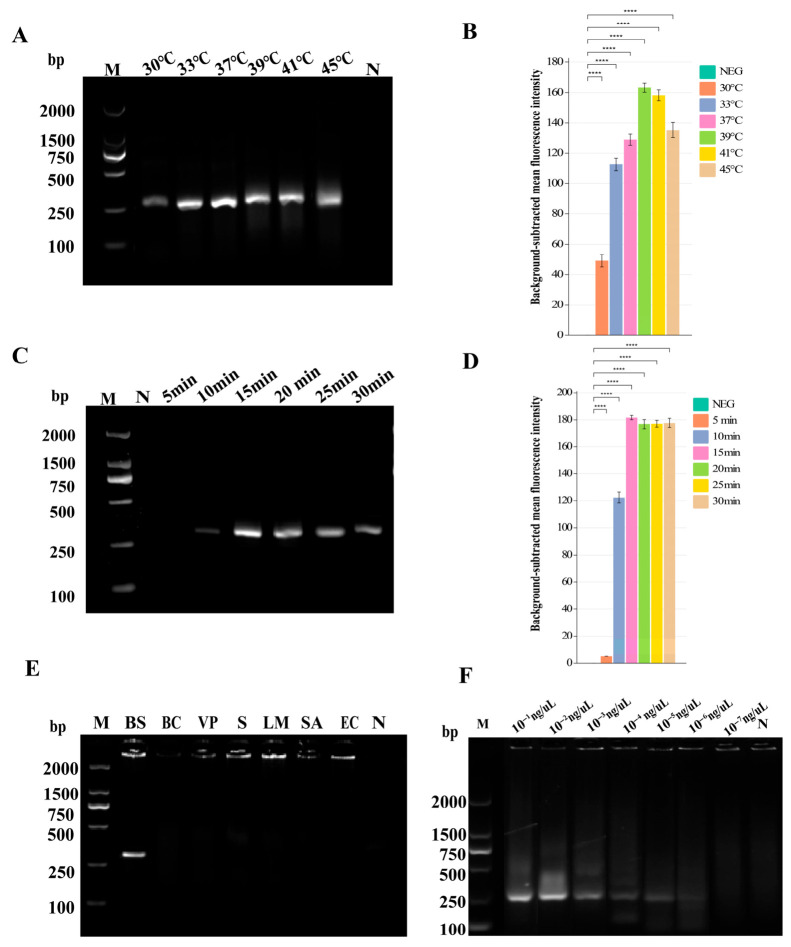
Optimization, specificity, and sensitivity of the RPA assay for BS detection. (**A**,**B**) Temperature optimization via gel electrophoresis and grayscale quantification. (**C**,**D**) Time optimization via gel electrophoresis and grayscale quantification. (**E**) Specificity test against common foodborne pathogens. (**F**) Sensitivity evaluation with serial DNA dilutions. Data are mean ± SD (n = 3), **** indicates a statistically significant difference with *p* < 0.0001. M: DL2000 DNA marker; N: Negative control.

The reaction temperatures were set at 30 °C, 33 °C, 37 °C, 39 °C, 41 °C, and 45 °C, with a fixed reaction time of 20 min. The optimal primer pair RPA-F2/R1 was used to amplify the genomic DNA of BS via RPA. Amplification efficiency was quantitatively evaluated by measuring the grayscale intensity of the target bands in AGE. As shown in Figure 3A,B, Quantitative grayscale analysis confirmed that the amplification efficiency at 39 °C was significantly higher than that at 37 °C (*p* < 0.05), and was comparable to that at 41 °C, with the most consistent and brightest band observed at 39 °C. Therefore, 39 °C was selected as the optimal reaction temperature for the RPA reaction in this method.

To optimize the RPA reaction time, the amplification was performed at the optimal temperature of 39 °C for 5, 10, 15, 20, 25, and 30 min, respectively. Amplification efficiency was quantitatively evaluated by measuring the grayscale intensity of the target bands in AGE. As shown in Figure 3C,D, no specific amplification was observed at 5 min, while clear target bands appeared from 10 min onwards. Quantitative grayscale analysis confirmed that the amplification efficiency increased significantly from 10 to 15 min (*p* < 0.05), and reached a plateau at 15 min, with no significant improvement in band brightness observed in subsequent time points (15–30 min, *p* > 0.05). Therefore, 15 min was selected as the optimal reaction time to balance amplification efficiency and detection speed.

According to the electrophoretogram Figure 3E, BS exhibited distinct amplification bands, while all other bacterial strains showed negative results, indicating no cross-reactivity between RPA amplification and non-target strains, and demonstrating high specificity.

Under the optimal reaction conditions of 39 °C and 15 min, the optimal primer pair was used to perform RPA-AGE detection on serially diluted genomic DNA of BS. A negative control without template was included, using ddH_2_O instead. As shown in Figure 3F, the minimum detectable concentration was 10^−5^ ng/μL, indicating high sensitivity of the RPA amplification.

### 3.3. Optimization of the RPA-CRISPR/Cas12a Detection System

**Figure 4:**
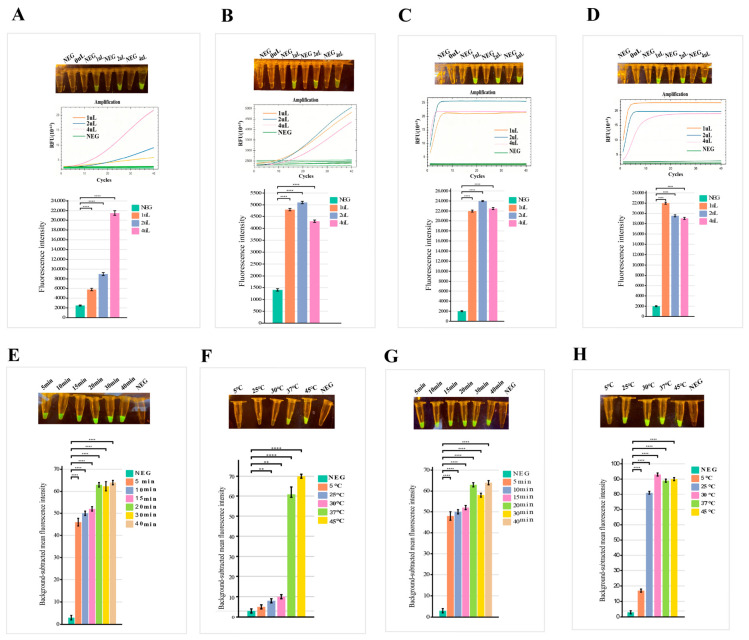
Optimization of the BS RPA-CRISPR/Cas12a detection system. Optimization of the CRISPR/Cas12a module and reaction conditions for crRNA2 and crRNA3. For crRNA2: (**A**) screening of different crRNA addition amounts; (**B**) screening of different Cas12a addition amounts; (**E**) reaction time optimization (5–40 min); (**F**) reaction temperature optimization (5–45 °C). For crRNA3: (**C**) screening of different crRNA addition amounts; (**D**) screening of different Cas12a addition amounts; (G) reaction time optimization (5–40 min); (H) reaction temperature optimization (5–45 °C). All fluorescence intensity data are presented as mean ± SD (n = 3). **** indicates a statistically significant difference with *p* < 0.0001.

As shown in Figure 4A,C, different volumes of crRNA (0 μL, 1 μL, 2 μL, and 4 μL) were added to the 20 μL detection system to optimize the crRNA dosage. The optimization was based on quantitative analysis of end-point fluorescence intensity with a no-template negative control included in all experiments. The results showed that the highest fluorescence signal and fastest reaction rate were achieved with 2 μL of crRNA, although visible fluorescence was also detected with 1 μL.

Based on the selected 2 μL crRNA, different volumes of Cas12a protein (0 μL, 1 μL, 2 μL, and 4 μL) were further evaluated. As illustrated in Figure 4B,D, quantitative end-point fluorescence analysis confirmed that the crRNA2 group exhibited optimal fluorescence intensity with 2 μL of Cas12a, while the crRNA3 group achieved the best performance with 1 μL of Cas12a, with all groups showing significantly higher signals than the negative control (****, *p* < 0.0001).

Using 2 μL crRNA and 1 μL Cas12a, the optimal reaction time for the CRISPR/Cas12a detection was evaluated at a preliminary temperature of 37 °C as shown in Figure 4E,G. A strong fluorescence signal was observed as early as 5 min, and the signal intensity increased significantly with prolonged reaction time, reaching a plateau at 10 min. Although there was some further improvement in signal intensity after 10 min, considering that the signal at 5 min was relatively weak, 10 min rather than 5 min was ultimately chosen as the optimal reaction time to ensure sufficient signal strength, reliable quantification, and good repeatability in practical detection.

Subsequently, under the conditions of 2 μL crRNA and 1 μL Cas12a with a 10 min reaction time, the fluorescence signal intensity was assessed at different temperatures in Figure 4F,H. The results showed that the crRNA2 group exhibited significantly stronger fluorescence at 37 °C, while the crRNA3 group achieved the highest signal intensity at 25 °C. Both groups demonstrated significantly higher signals than the negative control at all temperatures (****, *p* < 0.0001). Although crRNA3 exhibited slightly stronger fluorescence at 25 °C, 37 °C was ultimately selected as the optimal reaction temperature to ensure better compatibility with the preceding RPA amplification step (performed at 39 °C) and to maintain more robust and stable reaction performance under conditions close to room temperature, which is more favorable for potential on-site detection applications.

As shown in Figure 4, the fluorescence intensity of the crRNA3 group was consistently higher than that of the crRNA2 group across all experimental conditions throughout the optimization process. Considering detection cost, reaction efficiency, and signal stability comprehensively, the optimal detection conditions for the established RPA-CRISPR/Cas12a assay were determined as follows: 2 μL Cas12a reaction buffer, 1 μL HOLMES ssDNA Reporter (FAM), 1 μL Cas12a protein (1 μM), 2 μL crRNA (1 μM), 1 μL RPA product. Reaction temperature: 37 °C. Reaction time: 10 min.

### 3.4. Specificity of the RPA-CRISPR/Cas12a Detection System

**Figure 5:**
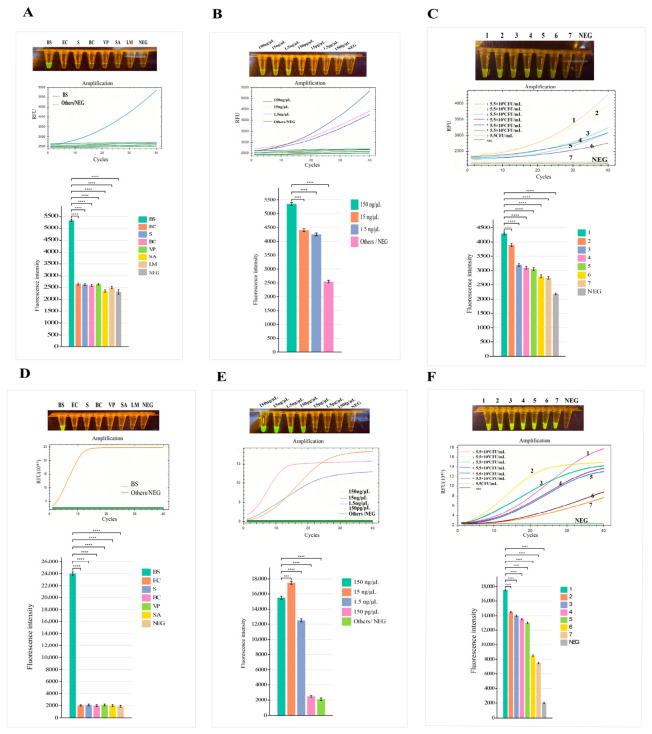
Sensitivity evaluation of the RPA-CRISPR/Cas12a assay for BS detection. (**A**,**D**) Specificity verification of crRNA2 and crRNA3, respectively (BS: *Bacillus subtilis*, EC: *Escherichia coli*, S: *Salmonella*, BC: *Bacillus cereus*, VP: *Vibrio parahaemolyticus*, SA: *Staphylococcus aureus*, LM: *Listeria monocytogenes*, NEG: negative control). (**B**,**E**) Genomic DNA sensitivity of crRNA2 and crRNA3, with detection limits of 1.5 ng/μL and 150 pg/μL, respectively. (**C**,**F**) Pure bacterial suspension sensitivity of crRNA2 and crRNA3. All experiments were performed in triplicate, data are presented as mean ± SD, and **** indicates a statistically significant difference with *p* < 0.0001.

Seven laboratory-preserved strains were used as templates, including BS, *Bacillus cereus*, *Escherichia coli*, *Salmonella*, *Vibrio parahaemolyticus*, *Staphylococcus aureus*, and *Listeria monocytogenes*. Amplification was performed using the *gyrB*-specific primer pair F2/R1. As shown in Figure 3E, target-specific amplification was observed only in BS. These seven strains were further analyzed using the RPA-CRISPR/Cas12a detection system. As shown in Figure 5A,D, a significant fluorescence signal was observed exclusively for BS, while signals for all non-BS strains remained at background levels. Notably, no cross-reactivity was observed even with the closely related *Bacillus cereus*, indicating the high specificity of both crRNA2 and crRNA3 for BS. These results confirm that the established RPA-CRISPR/Cas12a assay exhibits excellent species specificity and can accurately identify BS without interference from phylogenetically related Bacillus species or common foodborne pathogens.

### 3.5. Sensitivity of the RPA-CRISPR/Cas12a Detection System

To evaluate the analytical sensitivity of the RPA-CRISPR/Cas12a assay, the detection limit for BS genomic DNA was first determined. The genomic DNA of BS was serially 10-fold diluted, and each dilution was used as a template for RPA amplification, followed by CRISPR/Cas12a fluorescence detection. As shown in Figure 5B,E the crRNA2 group achieved a detection limit of 1.5 ng/μL genomic DNA, while the crRNA3 group exhibited significantly higher sensitivity, with a detection limit as low as 150 pg/μL genomic DNA. Statistical analysis confirmed that the fluorescence intensity of the crRNA3 group was significantly higher than that of the crRNA2 group at the same DNA concentration (****, *p* < 0.0001). The CRISPR/Cas12a reaction produced detectable fluorescence signals within 10 min, demonstrating that the established RPA-CRISPR/Cas12a method combines high detection sensitivity with rapid turnaround.

For the sensitivity testing of pure bacterial suspensions, BS cultures in the logarithmic growth phase were serially 10-fold diluted, ranging from 5.5 × 10^6^ CFU/mL to 5.5 CFU/mL. Genomic DNA was extracted from each dilution using the boiling method and subjected to RPA-CRISPR/Cas12a detection. As illustrated in Figure 5C, fluorescence intensity decreased progressively with the reduction in BS concentration. The crRNA3 group still produced a clear, positive fluorescence signal at a concentration as low as 5.5 CFU/mL, confirming its high sensitivity for viable bacterial detection.

### 3.6. Definition of Limit of Detection (LOD) and Detection of Bloated Soy Sauce Samples

**Figure 6:**
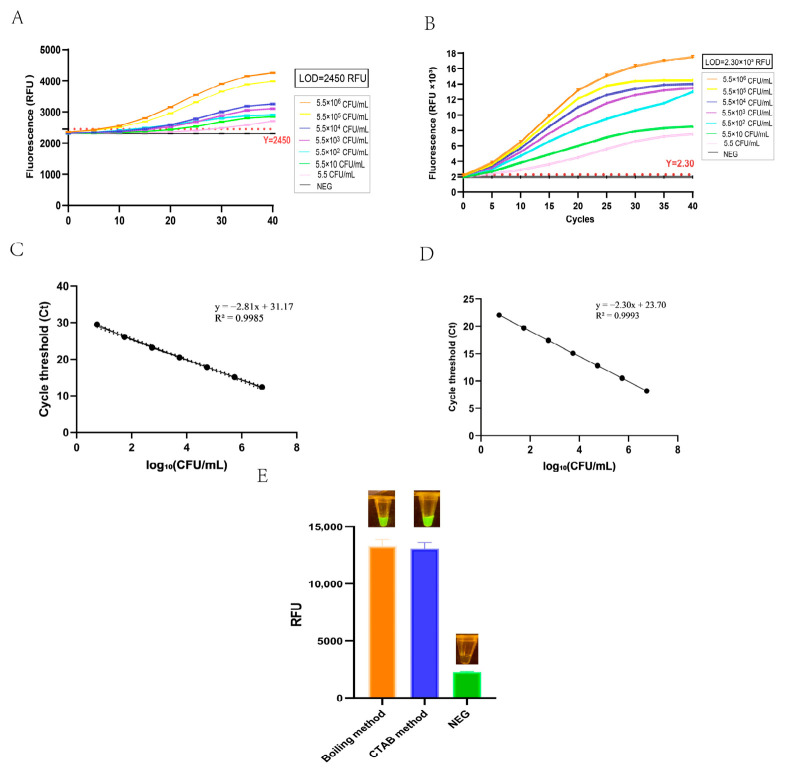
Real-time fluorescence curves and standard curves for the RPA-CRISPR/Cas12a assay. (**A**,**B**) Amplification curves with LOD thresholds calculated by the 3-sigma rule. (**C**,**D**) Linear standard curves for crRNA2 and crRNA3, with R^2^ > 0.998. (**E**) Practical detection of BS in swollen soy sauce samples using two DNA extraction methods. The orange bars represent the boiling method, and the blue bars represent the CTAB method.

In Figure 6A,C, the linear regression analysis between log_10_(CFU/mL) and Ct values yielded a standard curve with the equation y = −2.81x + 31.17 (R^2^ = 0.9985), indicating a strong positive correlation. Based on the defined LOD threshold (Mean_NEG_ + 3 × SD_NEG_ = 2.45 × 10^3^ RFU), the LOD of the crRNA2 based assay was determined to be 2.45 × 10^3^ RFU (corresponding to 55 CFU/mL).

In Figure 6B,D, a significant positive linear relationship was observed, with a high correlation coefficient y = −2.30x + 23.70 (R^2^ = 0.9993), indicating that the fluorescence intensity was reliably proportional to the target bacterial concentration in the range of 5.5–5.5 × 10^6^ CFU/mL. Using the 3σ rule, the LOD for the crRNA3 assay was calculated to be 2.30 × 10^3^ RFU (corresponding to5.5 CFU/mL), which was more sensitive than the crRNA2 group.

To evaluate the practical applicability of the established RPA-CRISPR/Cas12a assay, a naturally swollen soy sauce sample was tested using the crRNA3 system. As shown in Figure 6E, both the boiling and CTAB DNA extraction methods (Appendix A) produced strong fluorescence signals of approximately 13 × 10^3^ RFU, which were significantly higher than the LOD threshold of 2.30 × 10^3^ RFU determined by the 3σ rule. These results confirmed the presence of BS in the swollen soy sauce sample. No significant difference was observed between the two extraction methods, indicating that the simple boiling method is suitable for on-site detection. In summary, the developed assay meets the LOD requirement for BS detection in swollen soy sauce and provides a rapid and reliable on-site detection tool.

## 4. Discussion

In this study, an RPA-CRISPR/Cas12a assay targeting the *gyrB* gene was developed and optimized for the specific detection of BS [21]. Our results demonstrated that the *gyrB* gene served as a reliable target gene, enabling clear discrimination between BS and its phylogenetically closely related species *Bacillus cereus* as well as other common foodborne bacteria [8]. This result is consistent with previous studies indicating that the *gyrB* gene exhibits higher sequence variability among closely related *Bacillus* species than the 16S rRNA gene, thus supporting its suitability for species-specific detection [22].

During reaction optimization, crRNA dosage significantly affected Cas12a cleavage activity, and 2 μL of crRNA was identified as optimal for sufficient fluorescence signal. Similarly, Cas12a protein concentration influenced reaction efficiency, with 1 μL providing the best balance between signal intensity and detection cost. Although crRNA3 showed slightly higher fluorescence at 25 °C, 37 °C was chosen as the optimal temperature to ensure better compatibility with the preceding RPA reaction and more stable performance suitable for on-site application. While a detectable signal appeared as early as 5 min, 10 min was selected to ensure robust and repeatable signals rather than relying only on the minimal detectable time point. The established RPA-CRISPR/Cas12a system initiates strand cleavage at 37 °C and produces a detectable signal within 5 min, and the entire detection process including RPA amplification and CRISPR/Cas12a reaction can be completed within approximately 30 min [23]. This efficiency is considerably faster than conventional PCR or qPCR methods that typically require 60–90 min. The rapid detection speed, isothermal reaction conditions, and simple operation reduce dependence on large-scale precision instruments, making the method suitable for on-site rapid detection [24,25].

The sensitivity evaluation showed that the established assay exhibited a low detection limit toward BS in both genomic DNA and pure bacterial suspension samples. As a widely used probiotic, BS is required to maintain a viable count of at least 1 × 10^6^ CFU/g (mL) during shelf life according to the Regulations for the Application and Evaluation of Probiotic Health Foods (Trial) and related industrial standards [26]. The sensitivity achieved in this study fully meets the requirements for quantitative monitoring of probiotic products. For food safety monitoring, the regulatory limit for Bacillus cereus, a typical foodborne Bacillus pathogen, is usually set at 10^5^ CFU/g [27]. Although no exclusive national limit is specified for BS as a contaminant, the sensitivity of the present method is sufficient for the general monitoring of Bacillus species in foods [28].

However, it must be emphasized that the present study was conducted at the pure strain level using laboratory cultures. Nevertheless, the established assay has been successfully applied to detect BS in swollen soy sauce samples, demonstrating its practical feasibility in this typical spoiled food matrix. Matrix interference, background microbial competition, and DNA extraction efficiency may still affect actual detection performance in other real samples. Therefore, broader application in routine food monitoring still requires further evaluation using artificially contaminated or actual food samples. Future studies will focus on validating this method in more diverse food matrices, further assessing matrix effects, and establishing integrated protocols for on-site detection in food production and quality control.

## 5. Conclusions

In this study, an RPA-CRISPR/Cas12a assay targeting the *gyrB* gene was established for the rapid and visual detection of BS. The optimized crRNA3 with a TT-rich PAM sequence enabled efficient Cas12a trans-cleavage activity. Under the optimized conditions of 37 °C and 10 min, the assay achieved a detection limit of 150 pg/µL for genomic DNA and 5.5 CFU/mL for pure bacterial suspensions, respectively. The method showed good specificity against the tested strains, with no obvious cross-reactivity observed with Bacillus cereus or other common foodborne bacteria included in this study. These results demonstrate that the RPA-CRISPR/Cas12a system provides a promising, sensitive, and rapid approach for the detection of BS at the pure strain level.

Accordingly, further validation is warranted before broader application, including testing against a more comprehensive range of phylogenetically related *Bacillus* species, evaluation in spiked and more real food samples, and comparison with reference methods such as qPCR and culture-based standard procedures.

## Figures and Tables

**Table 1:** Sequences of primers and crRNAs.

Application	Primer Sequence (5′-3′)
RPA	F1 CGGAAGCGGCTATAAAGTATCCGGAGGATTAC
	F2 GTCGGTCGTAAACGCACTATCAACAGAG
	F3 ATTCACCGCCAAACCTATAAACGCGGAGTTC
	R1 CTTCACGTTTATCTTCAATCGTGATGTTTAC
	R2 CACGCGGTTGGCAAGCAGATCGTAATCATAC
crRNA	crRNA1 UAAUUUCUACUAAGUGUAGAUACCGUCACGGUGAACCGUCA
	crRNA2 UAAUUUCUACUAAGUGUAGAUCUCAGAAACAACCGAGUAUG
	crRNA3 UAAUUUCUACUAAGUGUAGAUGCAAGCAGAUCGUAAUCAUA

## Data Availability

The data that support the findings of this study are available on request to the corresponding authors.

## Supplementary Materials

The following supporting information can be downloaded at: https://www.mdpi.com/article/10.3390/foods15081419/s1, Figure S1: Schematic diagram (a) RPA amplification schematic (b) Cas12a cleavage schematic; Table S1: BS RPA primer information.

## Abbreviations

The following abbreviations are used in this manuscript:

RPA	Recombinase Polymerase Amplification e
CRISPR	Clustered Regularly Interspaced Short Palindromic Repeats
BS	*Bacillus subtilis*
BHI	Brain Heart Infusion
WHO	World Health Organization
FAO	Food and Agriculture Organization

## Appendix A. DNA Extraction by CTAB Method

In this study, a single sample of bloated-packaged soy sauce was used as the research subject. After initial enrichment culture of the microorganisms present in the sample, total DNA was extracted in parallel using both the boiling method (noted for its operational simplicity) and the CTAB method (recognized for its extraction efficiency).

The CTAB (cetyltrimethylammonium bromide) method is a classic protocol for extracting total DNA from complex samples, particularly suitable for soy sauce, which is rich in polysaccharides and polyphenols. The soy sauce sample was first collected by high-speed centrifugation and washed with PBS buffer to remove contaminants. The pellet was resuspended in preheated CTAB lysis buffer for cell lysis. Subsequently, an equal volume of chloroform-isoamyl alcohol (24:1) mixture was added for purification, and the supernatant was collected after centrifugation. DNA was recovered by isopropanol precipitation and further purified via washing with 70% ethanol. Finally, the dried pellet was resuspended in 100 μL of sterile water and stored at −20 °C.
