# Supplementary material for: Rapid Detection of Bacillus subtilis via RPA Combined with CRISPR/Cas12a
Source: Foods. 2026 Apr 18;15(8):1419. doi: 10.3390/foods15081419 (PMC13115003; doi:10.3390/foods15081419)
Supplement: Supplementary file 1 [file foods-15-01419-s001.zip › foods-4221081-supplementary.pdf]

Supplementary Materials

Rapid detection of Bacillus subtilis via RPA combined with CRISPR/Cas12a

Figure S1. Schematic diagram: (a) RPA amplification schematic and (b) Cas12a cleavage schematic.

Table S1. Detection of swelling in bag of soy sauce.

Since its introduction in 2006, recombinase polymerase amplification (RPA) has rapidly evolved and is regarded as a viable alternative to polymerase chain reaction (PCR). As illustrated in Figure S1(a), the principle of RPA is as follows: at a temperature range of 37–42°C, recombinase forms a complex with primers to identify and bind target DNA sequences, initiating strand displacement and DNA synthesis. Single-stranded binding (SSB) proteins stabilize the unwound DNA strands, while DNA polymerase extends the primers, achieving exponential amplification of the target sequence within 10 minutes. This method is characterized by high sensitivity, efficiency, and cost-effectiveness.

Cas12a (also known as Cpf1), a key effector protein in the CRISPR-Cas system, is widely used in gene editing. Unlike Cas9, it is guided by a single crRNA, recognizes a T-rich PAM sequence (located at the 5' end of the target site), and generates sticky-end breaks, which are more conducive to precise DNA insertion. As illustrated in Figure S1(b), the Cas12a–crRNA complex scans DNA, identifies the PAM sequence, and unwinds the local double-stranded DNA, allowing the crRNA to hybridize with the target strand. The RuvC nuclease domain of Cas12a is subsequently activated, cleaving the target DNA strand complementary to the crRNA first. Following this, Cas12a cleaves the exposed non-target strand. Moreover, after cleaving double-stranded DNA, Cas12a exhibits robust *trans*-cleavage activity, enabling non-specific cleavage of surrounding single-stranded nucleic acids. This property has been extensively utilized in nucleic acid detection technologies.

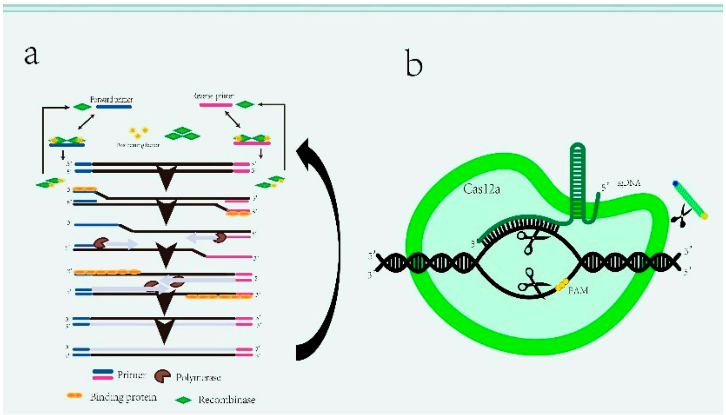

Figure S1. Schematic diagram: (a) RPA amplification schematic and (b) Cas12a cleavage schematic.

Table S1. Bacillus subtilis RPA primer information.

Primer name	Primer sequence（5' -3'）	Fragment length
gyrB-F1	CGGAAGCGGCTATAAAGTATCCGGAGGATTAC	328bp
gyrB-R1	CTTCACGTTTATCTTCAATCGTGATGTTTAC	
gyrB-F2	GTCGGTCGTAAACGCACTATCAACAGAG	272bp
gyrB-R1	CTTCACGTTTATCTTCAATCGTGATGTTTAC	
gyrB-F3	ATTCACCGCCAAACCTATAAACGCGGAGTTC	214bp
gyrB-R1	CTTCACGTTTATCTTCAATCGTGATGTTTAC	
gyrB-F1	CGGAAGCGGCTATAAAGTATCCGGAGGATTAC	259bp
gyrB-R2	CACGCGGTTGGCAAGCAGATCGTAATCATAC	
gyrB-F2	GTCGGTCGTAAACGCACTATCAACAGAG	214bp
gyrB-R2	CACGCGGTTGGCAAGCAGATCGTAATCATAC	
gyrB-F3	ATTCACCGCCAAACCTATAAACGCGGAGTTC	153bp
gyrB-R2	CACGCGGTTGGCAAGCAGATCGTAATCATAC	
